# Pioglitazone Protects Compression-Mediated Apoptosis in Nucleus Pulposus Mesenchymal Stem Cells by Suppressing Oxidative Stress

**DOI:** 10.1155/2019/4764071

**Published:** 2019-11-22

**Authors:** Yiqiang Hu, Liang Huang, Min Shen, Yunlu Liu, Guohui Liu, Yongchao Wu, Fan Ding, Kaige Ma, Wentian Wang, Yanbin Zhang, Zengwu Shao, Xianyi Cai, Liming Xiong

**Affiliations:** ^1^Department of Orthopedics, Union Hospital, Tongji Medical College, Huazhong University of Science and Technology, Wuhan 430022, China; ^2^Department of Cardiology, Union Hospital, Tongji Medical College, Huazhong University of Science and Technology, Wuhan 430022, China; ^3^Department of Hematology, Tongji Hospital, Tongji Medical College, Huazhong University of Science and Technology, Wuhan 430030, China; ^4^Department of Orthopaedic Surgery, The First People's Hospital of Jingmen, Jingmen 448000, Hubei, China

## Abstract

Excessive compression, the main cause of intervertebral disc (IVD) degeneration, affected endogenous repair of the intervertebral disc. Pioglitazone (PGZ) is the agonist of peroxisome proliferator-activated receptor *γ*, which has been widely used in the treatment of diabetes mellitus. The present study aim at investigating whether pioglitazone has protective effects on compression-mediated cell apoptosis in nucleus pulposus mesenchymal stem cells (NP-MSCs) and further exploring the possible underlying mechanism. Our results indicated that the isolated cells satisfied the criteria of MSC stated by the International Society for Cellular Therapy. Besides, our research revealed that pioglitazone could protect cell viability, cell proliferation of NP-MSCs and alleviated the toxic effects caused by compression. The actin stress fibers was suppressed obviously under compression, and pioglitazone alleviated the adverse outcomes. Pioglitazone exerted protective effects on compression-induced NP-MSCs apoptosis according to annexin V/PI double-staining and TUNEL assays. Pioglitazone suppressed compression-induced NP-MSCs oxidative stress, including decreasing compression-induced overproduction of reactive oxygen species (ROS) and malondialdehyde (MDA), and alleviated compression-induced mitochondrial membrane potential (MMP) decrease. Ultrastructure collapse of the mitochondria exhibited a notable improvement by pioglitazone in compression-induced NP-MSCs according to transmission electron microscopy (TEM). Furthermore, the molecular results showed that pioglitazone significantly decreased the expression of apoptosis-associated proteins, including cyto.cytochrome c, Bax, cleaved caspase-9, and cleaved caspase-3, and promoted Bcl-2 expression. These results indicated that pioglitazone alleviated compression-induced NP-MSCs apoptosis by suppressing oxidative stress and the mitochondrial apoptosis pathway, which may be a valuable candidate for the treatment of IVD degeneration.

## 1. Introduction

Intervertebral disc (IVD) degeneration is a major cause for low back pain (LBP), which has become a global social problem affecting human life and causes a great economic burden on governments [1–3]. At present, the main therapeutic regimens for IVD degeneration are conservative therapy and surgical decompression of the spine, aimed at alleviating the pain and returning patients to work [4]. However, neither of the strategies is effective for treating IVD degeneration. At present, stem cell therapy has increasingly become a new therapy strategy for the repair of IVD degeneration. Mesenchymal stem cells (MSCs) can differentiate into NP-like cells and promote extracellular matrix (ECM) synthesis, which have exhibited great potential for treating IVD degeneration. Recently, MSCs were found existing in NP tissue, and these NP-MSCs efficiently repaired NP tissue [5, 6]. Many researches showed that the loss of IVD-MSCs number and function was closely related to IVD degeneration [7, 8].

Mechanical stress is an important inducement of IVD degeneration. Generally, the IVD experiences various mechanical loads in daily activities. Chou et al. demonstrated that fluid-induced shear stress changed the expressions of ECM and metalloproteinase genes in IVD cells [9]. Also, under this compression microenvironment, IVD stem cells also suffered from excessive cell death, which made it tough to maintain the number and viability of stem cells in IVDs [10]. Previously, we reported that compression decreased the number and viability of IVD cells, which contributed to IVD degeneration [11]. Recently, we found that compression induced substantial NP-MSCs death [12]. Considering all of the above, seeking an effective pharmaceutical to reduce the NP-MSCs death will be a promising therapy for IVD degeneration.

Pioglitazone, considered as a peroxisome proliferator-activated receptor *γ* activator, is used to treat diabetes mellitus [13]. It plays potential beneficial roles in inflammation, fat distribution, and lipid and protein metabolism [14–16]. Shen et al. reported that pioglitazone protected pancreatic cells against palmitic acid-induced cytotoxicity through antioxidative stress [17]. Recently, researchers reported that pioglitazone alleviated cell apoptosis induced by TNF-*α* or stress in endothelial cells by altering the Bcl-2 expression and caspase-3 activation [18, 19]. In addition, pioglitazone inhibited advanced glycation end product-induced chondrocyte apoptosis, having a potential therapeutic ability for AGE-induced mouse osteoarthritis with diabetes [20]. However, the influence of pioglitazone on compression-induced NP-MSCs death remains unknown.

Apoptosis as the programmed cell death plays a vital role in eliminating the injured or nonessential cells [21]. Apoptotic signals initiated the mitochondrial dysfunction pathway and caused an increase of intracellular ROS levels, which made oxidative stress occur [22, 23]. Excessive ROS production led to oxidative damage and cell death [24]. ROS could directly impair the mitochondrial function, which then decreased the MMP and released the cytochrome c into the cytosol [25]. The release of cytochrome c was inhibited by the antiapoptotic members of the Bcl-2 family and stimulated by the proapoptotic members, such as Bax. Cytochrome c promoted the formation of the apoptosome by recruiting caspase-9, which then induced caspase-3 activation and triggered the caspase cascade, eventually causing the cell apoptotic death [26–28].

In this research, we used different methods to evaluate the protective role of pioglitazone on compression-induced NP-MSCs apoptosis as well as oxidative stress levels. In addition, we evaluated the role of the mitochondrial pathway in pioglitazone protecting compression-induced NP-MSCs apoptosis by detecting apoptosis-related proteins and mitochondrial function.

## 2. Material and Methods

### 2.1. Isolation and Culture of NP-MSCs

All experiments in this study were approved by the Clinical Research Ethics Committee of Tongji Medical College, Huazhong University of Science and Technology. The nucleus pulposus samples were obtained from patients undergoing discectomy for degenerative disc disease. NP-MSCs were isolated and incubated as we reported in our previous studies [12]. In brief, after being washed with normal saline, NP tissues were cut into small pieces and digested with 0.25% type II collagenase for 8 h. And then, the digested tissues were cultured in MSC complete medium (Cyagen, CA, USA) at 37°C and 5% CO_2_. The culture medium was changed every three days. The NP-MSCs were purified and cultured from the primary cells. They were seeded in the appropriate culture plates for subsequent experiments when second-passage NP-MSCs reached 80%-90% confluence.

### 2.2. Surface Marker Identification of NP-MSCs

NP-MSCs were collected and washed with phosphate-buffered saline (PBS). The cells were resuspended and incubated with a solution of antibodies of CD73, CD90, CD105, CD34, and HLA-DR at 37°C for 30 min. After being incubated with the antibodies, the cells were centrifuged at 1500 rpm for 5 min. The labeled cells were rinsed and then marked by secondary antibodies at 37°C for 30 min. Cells were washed twice and resuspended in 200 *μ*l PBS. The labeled cells were examined by flow cytometry (Becton Dickinson, Franklin Lakes, NJ, USA).

### 2.3. Multilineage Differentiation

To assess the multilineage differentiation potential of NP-MSCs, the osteogenic, adipogenic, and chondrogenic differentiations were induced. In brief, NP-MSCs were seeded in culture plates with normal medium. When NP-MSCs reached the appropriate confluence, the conditional medium was changed with human mesenchymal stem cell osteogenic, adipogenic, and chondrogenic differentiation media (Cyagen, CA, USA) respectively according to the manufacturer's protocol. The osteogenic, adipogenic, and chondrogenic differentiation potential of NP-MSCs was assessed by Alizarin Red, Oil Red O, and Alcian blue staining after 21 days, 30 days, and 28 days, respectively. The results were detected by a light microscope (Olympus, Japan).

### 2.4. Colony Forming Assay

The colony forming assay was detected with NP-MSCs seeded in 6-well plates at 1000 cells/well. After being cultured 10 days, the NP-MSCs were fixed with 4% paraformaldehyde for 20 min. The cells were washed two times with PBS and stained with 1% crystal violet stain (Solarbio, China) for 30 min, then washed two times with PBS. Colonies of NP-MSCs were observed.

### 2.5. Application of a Compression Apparatus on NP-MSCs

NP-MSCs were cultured in a pressure apparatus to simulate in vivo conditions as we previously described [1, 11, 12, 29–31]. To study the effects of pioglitazone on compression-exposed NP-MSCs, cells were treated with different concentrations of pioglitazone (0 *μ*M, 10 *μ*M, 20 *μ*M, 50 *μ*M, and 100 *μ*M) and exposed to 1.0 MPa compression for 36 h. Then, they were used for subsequent experiments. Control group cells (con) served as a control group in this study.

### 2.6. CCK-8 Assay

NP-MSCs viability was assessed by Cell Counting Kit-8 (CCK-8, Dojindo, Japan) assay as described previously [3]. In brief, the cell densities were 5 × 10^3^ cells/well for CCK-8. After being treated, 100 millilitres (ml) of DMEM/F-12 containing 10 ml of CCK-8 solution was added to each well, and the 96-well plates were incubated for 4 h at 37°C. The absorbance was determined with a spectrophotometer at 450 nm (BioTek, Winooski, VT, USA).

### 2.7. EdU Incorporation Assay

The effect of pioglitazone on compression-mediated NP-MSCs proliferation was evaluated by 5-ethynyl-2′-deoxyuridine (EdU) incorporation assay (Ribobio, China). After being treated with pioglitazone or compression, NP-MSCs were stained with the EdU incorporation assay according to the manufacturer's instructions. After being washed for three times with PBS, EdU staining of NP-MSCs was detected in the dark by a fluorescence microscope (Olympus, Japan).

### 2.8. Rhodamine-Phalloidin Staining

Rhodamine-labelled phalloidin (Cytoskeleton, Denver, CO) was used to stain the actin filaments in NP-MSCs. Briefly, after being treated, the cells were fixed in 4% formaldehyde for 10 min at room temperature and permeabilized with 0.3% Triton X-100 in PBS for 15 min. The cells were incubated with rhodamine-phalloidin (100 nM) in the dark for 30 min. Then, the cells were washed two times and incubated with DAPI staining (Boster, China) for 5 min in the dark. After being washed two times with PBS, the cells were observed and photographed by using a confocal microscopy (Nikon A1, Japan).

### 2.9. Lactate Dehydrogenase Release Assay

Release of lactate dehydrogenase (LDH) was used to assess the effect of pioglitazone on compression-exposed NP-MSCs. Briefly, after being treated, NP-MSCs were incubated with the LDH assay according to the manufacturer's protocol (Beyotime, China). The absorbance at 490 nm was measured using a microplate reader (Biotek, Winooski, VT, USA).

### 2.10. Live/Dead Assay

We used calcein-AM/PI (Dojindo, Japan) to evaluate the effect of pioglitazone on compression-treated NP-MSCs. After being treated, NP-MSCs were washed two times and then incubated with calcein-AM and propidium iodide (PI) in the dark for 20 min at 37°C. Live cells displayed green fluorescence (calcein-AM-positive) while the dead cells displayed red fluorescence (PI-positive) under the fluorescence microscope. The live/dead cells were detected in the dark by a fluorescence microscope (Olympus, Japan).

### 2.11. Annexin V/PI Staining

Cell apoptosis was quantified by annexin V/PI staining according to the manufacturer's instructions (KeyGen Biotech, China). After being treated, the NP-MSCs were collected and washed, then a binding buffer was used to resuspend NP-MSCs. 5 *μ*l annexin V and 5 *μ*l PI were used to label NP-MSCs for 15 min, and the cells were analyzed by flow cytometry (Becton Dickinson, Franklin Lakes, NJ, USA).

### 2.12. TUNEL Staining

We used TUNEL (terminal deoxynucleotidyl transferase-mediated dUTP nick end labeling) staining to evaluate the cell apoptosis. After being fixed in 4% paraformaldehyde for 25 min at room temperature, NP-MSCs were permeabilized with 0.1% TritonX-100 for 10 min. According to the protocol of the manufacturer (Roche, Germany), the cells were washed with PBS, then incubated with staining at 37°C in the dark. Apoptotic changes were observed under the inverted fluorescence microscope (Olympus, Japan).

### 2.13. Measurement of ROS Production

Intracellular ROS levels were measured by DCFH-DA assay (Nanjing Jiancheng, China). After being treated, NP-MSCs were collected and washed in PBS, then incubated with DCFH-DA at 37°C for 20 min. The ROS levels were measured using by flow cytometry (Becton Dickinson, Franklin Lakes, NJ, USA).

### 2.14. MDA Assay

The MDA content, which reflect oxidation levels, was quantified using a lipid peroxidation MDA assay kit (Beyotime, China) according to the manufacturer's instructions. The samples and standards were prepared and the OD value was measured at 532 nm. MDA concentrations (nmol/ml) were expressed as nmol/mg protein.

### 2.15. JC-1 Staining

MMP was measured by using a JC-1 (“5,5′,6,6′-tetrachloro-1,1′,3,3′-tetraethyl-imidacarbocyanine iodide”) staining kit (Beyotime, China). Briefly, NP-MSCs from every group were collected and washed with PBS, then resuspended in 1 ml culture medium which contained 0.5 ml JC-1 staining fluid. NP-MSCs were incubated with JC-1 staining fluid for 20 min at 37°C with 5% CO_2_. The cells were washed twice and resuspended in 0.5 ml staining buffer. The MMP of each group was measured by flow cytometry and expressed as the ratio of red/green fluorescence intensity (Becton Dickinson, Franklin Lakes, NJ, USA). Furthermore, MMP was determined by a fluorescence microscope (Olympus, Japan).

### 2.16. Transmission Electron Microscopy

Transmission electron microscopy (TEM) was used to examine changes in the ultrastructure of NP-MSCs as described previously [31]. Briefly, NP-MSCs were harvested and washed with PBS. After being pelleted by centrifugation at 1500 rpm for 5 min, the cells were fixed with 2.5% glutaraldehyde for 12 h. Then, the cells were dehydrated in ethanol and infiltrated and embedded. Ultrathin sections were stained with uranyl acetate and examined under transmission electron microscope (FEI Company, USA).

### 2.17. Western Blot Analysis

The cells were lysed by using a western and IP cell lysis kit. After NP-MSCs were treated with 100 *μ*M pioglitazone or 1.0 MPa compression, apoptosis-related proteins were detected by western blotting. Also, to determine the cytochrome c of cytosol (cyto.cytochrome c), the cytosol protein of cells was isolated according to the manufacturer's instructions of the Cell Mitochondria Isolation Kit (Beyotime, China). The protein concentration was measured by using the BCA protein assay kit (Beyotime, China). Equal protein amounts (30 *μ*g) were resolved on 10%-12% SDS-PAGE gel and then transferred onto PVDF membranes (Millipore, Burlington, MA, USA), then the membranes were blocked by 5% nonfat milk and then incubated overnight at 4°C with primary antibodies against cytochrome c (1 : 1000, Abcam, USA, ab90529), Bcl-2 (1 : 1000, Abcam, USA, ab59348), Bax (1 : 1000, Abcam, USA, ab32503), cleaved caspase-9 (1 : 1000, Abcam, USA, ab2324), and cleaved caspase-3 (1 : 1000, Abcam, USA, ab2302) overnight at 4°C. After being washed with TBST for three times, the membranes were incubated with secondary antibodies for 1 h at room temperature. Finally, the enhanced chemiluminescence method was used to visualize the proteins.

### 2.18. Statistical Analysis

GraphPad Prism (GraphPad Software Inc.; La Jolla, CA, USA) was used for statistical analysis. Values are presented as the mean ± standard deviation (SD) from at least three independent experiments. Student's *t*-test and one-way analysis of variance (ANOVA) were used to perform the statistical significance of the differences between two groups and multiple groups, respectively. Bonferroni's post hoc test was used to determine the source of the observed differences. *P* < 0.05 was considered as statistically significant.

## 3. Results

### 3.1. Identification of NP-MSCs

**Figure 1:**
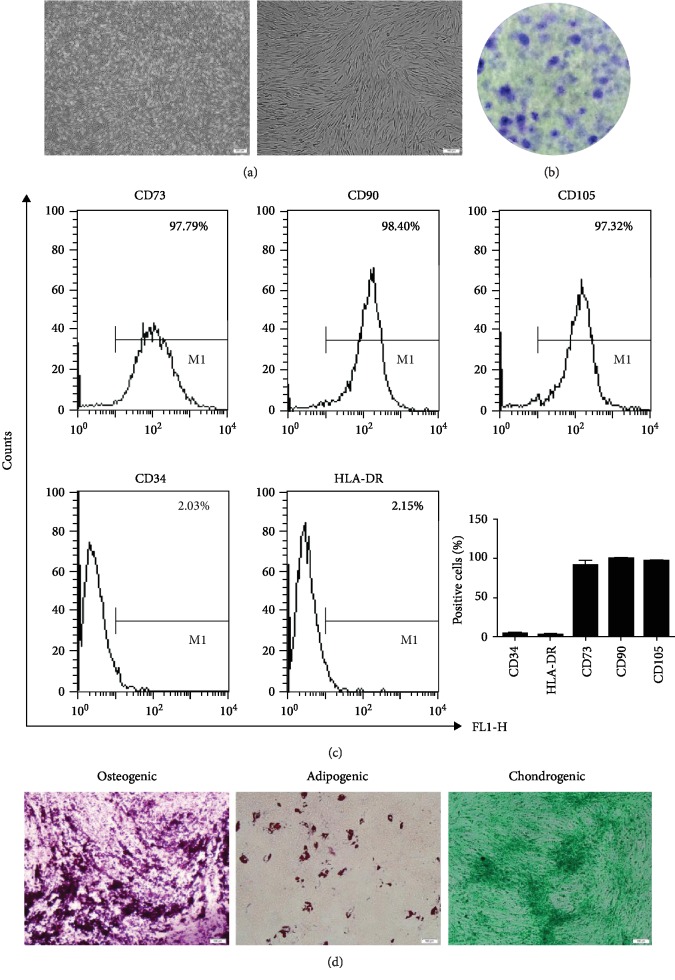
Identification of NP-MSCs. (a) The growth state and morphology of the cells were observed under an optical microscope (40x and 100x). (b) A colony-forming assay was used to observe NP-MSCs-formed homogeneous colonies (1x). (c) Flow cytometry was used to detect the surface markers on the cells, including CD34, CD73, CD90, CD105, and HLA-DR. Quantitative analysis of positive cells in NP-MSCs. (d) Alizarin Red staining, Oil Red O staining, and Alcian blue were used to evaluate osteogenic, adipogenic, and chondrogenic differentiation of NP-MSCs, respectively (100x).

NP-MSCs were isolated and cultured from IVD. We observed that these cells displayed vortex-style adherent growth and showed a long spindle shape (Figure 1(a)). These cells formed homogeneous colonies (Figure 1(b)). The surface markers on the cells were detected by flow cytometry. NP-MSCs highly expressed surface markers, including CD73, CD90, and CD105. The positive rates of those were more than 95%. NP-MSCs expressed less CD34 and HLA-DR (Figure 1(c)). Furthermore, we assessed the multilineage differentiation potential of NP-MSCs. NP-MSCs formed many visible calcium deposits by Alizarin Red staining after osteogenic induction. After adipogenic differentiation, oil droplets were observed in NP-MSCs by Oil Red O staining. Also, these cells largely produced sulfated proteoglycans after chondrogenic induction by Alcian blue (Figure 1(d)). The results above proved that the isolated cells satisfied the criteria of MSC stated by ISCT. Thus, we successfully isolated and cultured NP-MSCs, and the second-generation cells were used in this study.

### 3.2. Pioglitazone Alleviated the Inhibitory Effect of Compression on Cell Viability and Cell Proliferation

**Figure 2:**
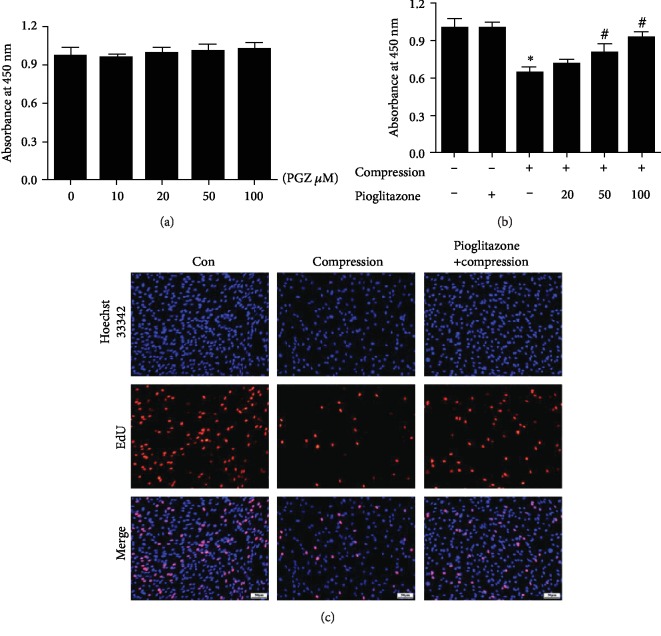
Effects of pioglitazone on NP-MSCs viability and proliferation. (a) The CCK-8 assay was used to evaluate the cell viability with various concentrations of pioglitazone (0, 10, 20, 50, and 100 *μ*M) on NP-MSCs. (b) The CCK-8 assay was used to evaluate the effect of pioglitazone on compression-mediated NP-MSCs viability. Con served as a control group. Data are presented as the mean ± SD of three independent experiments. ^∗^*P* < 0.05 versus the control group; ^#^*P* < 0.05 versus the compression group. (c) Representative micrographs of EdU staining by fluorescence microscopy. The red fluorescence indicates EdU-positive cells (200x).

CCK-8 assay was performed to determine NP-MSCs viability. The results of CCK-8 indicated that pioglitazone had no cytotoxicity on NP-MSCs (Figure 2(a)). We found that compression significantly decreased cell viability. To investigate the protective role of pioglitazone on NP-MSCs, we treated NP-MSCs with different concentrations of pioglitazone. We found that pioglitazone significantly alleviated the inhibitory effect of compression on cell viability. And the protective effect was obvious at the doses of 100 *μ*M (Figure 2(b)). Thus, 100 *μ*M pioglitazone was used in the subsequent experiments. In addition, we used the EdU incorporation assay to detect cell proliferation. The results indicated that the number of EdU-positive cells (red fluorescence) in pioglitazone groups was more than that in the compression groups (Figure 2(c)). These proved that pioglitazone remarkably alleviated the inhibitory effects of compression on cell proliferation.

### 3.3. Pioglitazone Protected against Compression-Induced Cytotoxicity in NP-MSCs

**Figure 3:**
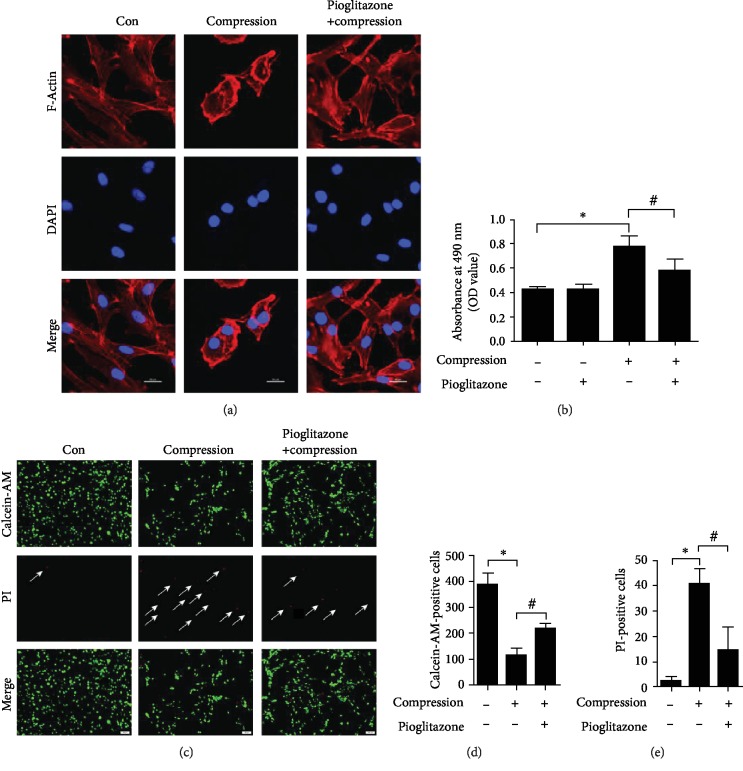
Pioglitazone protected against compression-induced cytotoxicity in NP-MSCs. (a) Rhodamine-phalloidin was used to detect the actin filaments of NP-MSCs. The red fluorescence showed the actin (1000x). (b) The histogram shows the release of LDH. (c) Calcein-AM/PI dye was used to evaluate NP-MSCs damage under a fluorescence microscope. The green fluorescence indicates live cells, and the red fluorescence indicates dead cells (100x). (d) Quantitative analysis of calcein-AM-positive cells. (e) Quantitative analysis of PI-positive cells. Data are presented as the mean ± SD of three independent experiments. ^∗^*P* < 0.05 versus the control group; ^#^*P* < 0.05 versus the compression group.

Rhodamine-phalloidin was used to detect the actin filaments of NP-MSCs. The results displayed that compression obviously broke the actin stress fibers and pioglitazone rescued the adverse outcomes caused by compression (Figure 3(a)). Subsequently, we detected the release of LDH. The results demonstrated that compression significantly enhanced the release of LDH compared with the control group. Pioglitazone significantly reversed the release of LDH induced by compression (Figure 3(b)). To further assess the protective effects of pioglitazone on compression-induced cytotoxicity in NP-MSCs, we used calcein-AM/PI assay to quantify the numbers of live/dead cells under a fluorescence microscope. We found that live cells (green fluorescence) were more in the pioglitazone groups while dead cells (red fluorescence) were more frequent in the compression groups (Figures 3(c)–3(e)). Those results demonstrated that pioglitazone protected against compression-induced cell cytotoxicity in NP-MSCs.

### 3.4. Protective Effects of Pioglitazone on Compression-Induced Apoptosis of NP-MSCs

**Figure 4:**
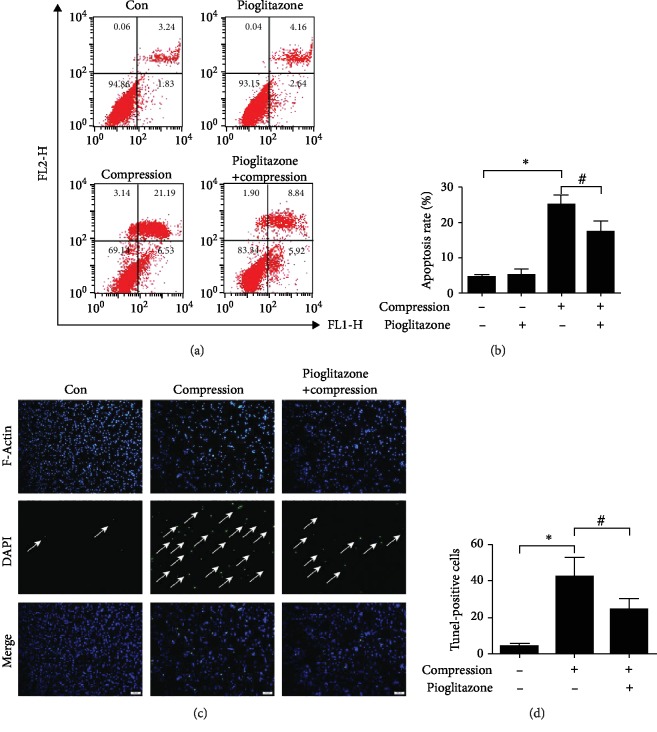
Effects of pioglitazone on NP-MSCs apoptosis. (a) Flow cytometric analysis was used to detect cell apoptosis by annexin V/PI staining. (b) Quantitative analysis of the apoptotic NP-MSCs rate. (c) The TUNEL staining was used to investigate apoptotic changes under a fluorescence microscope (100x). (d) Quantitative analysis of TUNEL-positive cells. Data are presented as the mean ± SD of three independent experiments. ^∗^*P* < 0.05 versus the control group; ^#^*P* < 0.05 versus the compression group.

To explore the protective effects of pioglitazone on compression-induced apoptosis, we used annexin V/PI double-staining to examine the apoptosis of NP-MSCs. The results of flow cytometry showed that compression induced NP-MSCs apoptosis. Pioglitazone can significantly protect compression-induced apoptosis of NP-MSCs (Figures 4(a) and 4(b)). To further examine the protective effects of pioglitazone, the TUNEL staining was used to investigate apoptotic changes under a fluorescence microscope. TUNEL staining revealed that the TUNEL-positive cells were obviously decreased in the pioglitazone group compared with the compression group (Figures 4(c) and 4(d)). These results showed that pioglitazone exerted protective effects on compression-induced NP-MSCs apoptosis.

### 3.5. Pioglitazone Reduced Compression-Induced Oxidative Stress in NP-MSCs

**Figure 5:**
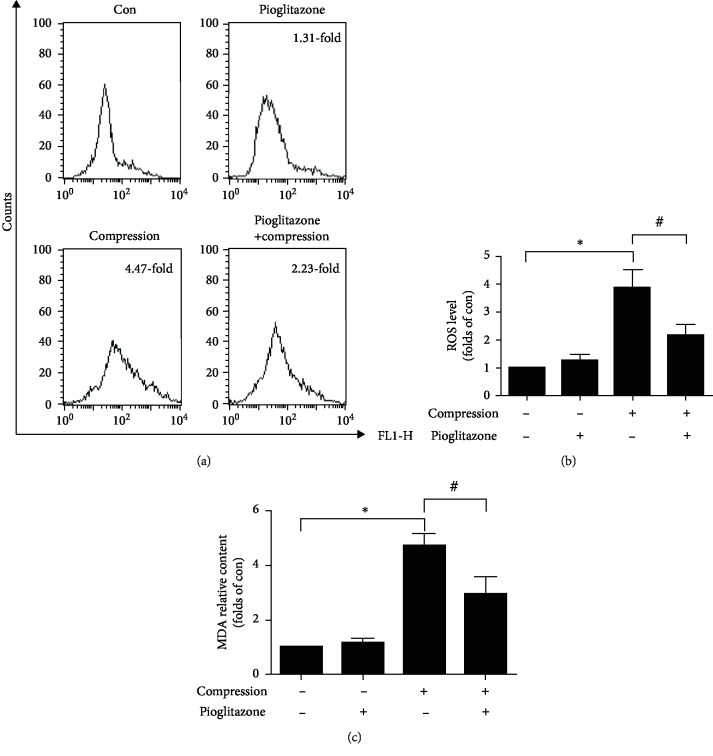
Effect of pioglitazone on compression-induced oxidative stress in NP-MSCs. (a) Flow cytometric analysis was performed to detect intracellular ROS production in NP-MSCs. (b) Quantitative analysis of intracellular ROS production in NP-MSCs. (c) A lipid peroxidation MDA assay kit was used to assess MDA levels. Data are presented as the mean ± SD of three independent experiments. ^∗^*P* < 0.05 versus the control group; ^#^*P* < 0.05 versus the compression group.

Increased levels of oxidative stress caused mitochondrial dysfunction and resulted in cell apoptosis. To investigate the effects of pioglitazone on oxidative stress of NP-MSCs, we used DCFH-DA fluorescent probes to detect ROS production to evaluate oxidative stress levels of NP-MSCs. We found that compression significantly increased intracellular ROS production as compared with the control group. Pioglitazone can significantly reduce compression-induced ROS production (Figures 5(a) and 5(b)). In addition, we also assessed the oxidative stress level by the lipid peroxidation MDA assay kit. The data showed that pioglitazone significantly reduced MDA which was high in the compression group (Figure 5(c)). These results showed that pioglitazone alleviated compression-induced oxidative stress levels in NP-MSCs.

### 3.6. Pioglitazone Alleviated Compression-Induced Mitochondrion Damage in NP-MSCs

**Figure 6:**
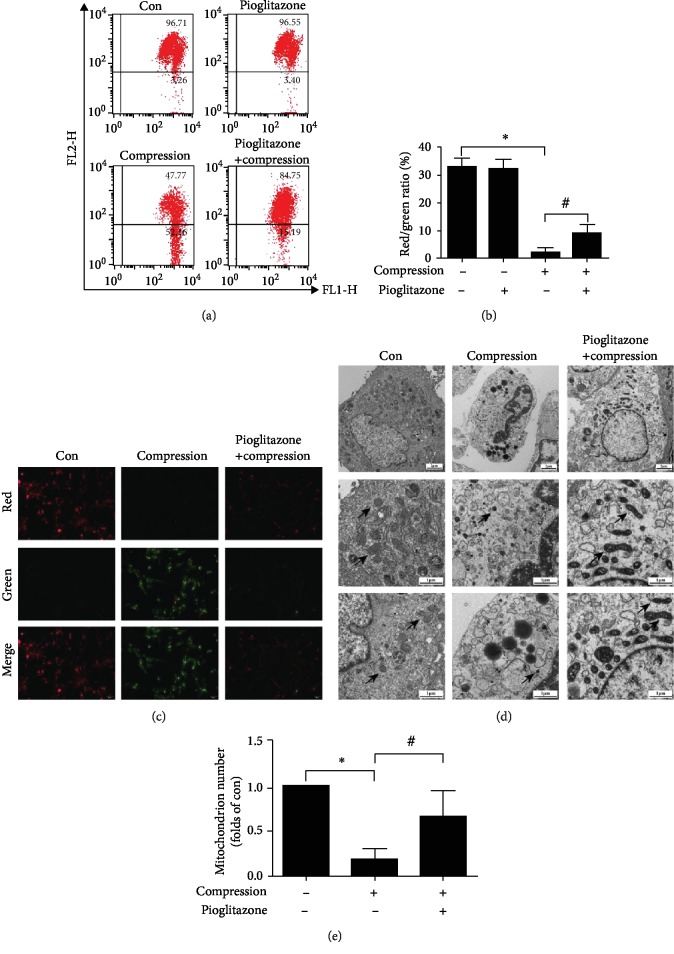
Effect of pioglitazone on compression-induced mitochondrion damage in NP-MSCs. (a) JC-1 staining was used to detect MMP in NP-MSCs by flow cytometric analysis. (b) Quantitative analysis of MMP in NP-MSCs. (c) MMP of NP-MSCs was observed under a fluorescence microscope (200x). (d) The cell nucleus and mitochondria of NP-MSCs were observed by TEM in three groups. Mitochondria were displayed as indicated by the arrowheads (1700x and 5000x). (e) Quantitative analysis of mitochondrion numbers in NP-MSCs. Data are presented as the mean ± SD of three independent experiments. ^∗^*P* < 0.05 versus the control group; ^#^*P* < 0.05 versus the compression group.

Mitochondrion damage is an important factor in the mitochondrial apoptotic pathway. When MMP loss occurred in mitochondrion damage, JC-1 shifted from JC-1 aggregates (red fluorescence) to JC-1 monomers (green fluorescence). To evaluate the protective effects of pioglitazone on compression-induced mitochondrion damage of NP-MSCs, we operated JC-1 staining to detect MMP in NP-MSCs by flow cytometric analysis. We found that compression reduced the red/green ratio in NP-MSCs and pioglitazone significantly increased the ratio of red/green compared with the compression group (Figures 6(a) and 6(b)). Also, we used a fluorescence microscope to observe the protective effects of pioglitazone. In the compression group, NP-MSCs exhibited less red fluorescence but more green fluorescence, while pioglitazone could increase the red fluorescence and reduce the green fluorescence (Figure 6(c)). Meanwhile, we applied TEM to intuitively observe the ultrastructure of NP-MSCs. When NP-MSCs were exposed to compression, the nucleus shrunk and the apoptotic corpuscles appeared. Furthermore, the numbers of mitochondria were reduced obviously, and a big lipid droplet appeared in the compression group. Fortunately, pioglitazone can significantly alleviate the damage in the ultrastructure of NP-MSCs. Specifically, the nucleus was relatively normal when compared with that of the compression group, and the numbers and the morphology of mitochondria were improved (Figure 6(d)).

### 3.7. Effects of Pioglitazone on Mitochondrial Pathway Apoptosis-Related Protein Expression under Compression in NP-MSCs

**Figure 7:**
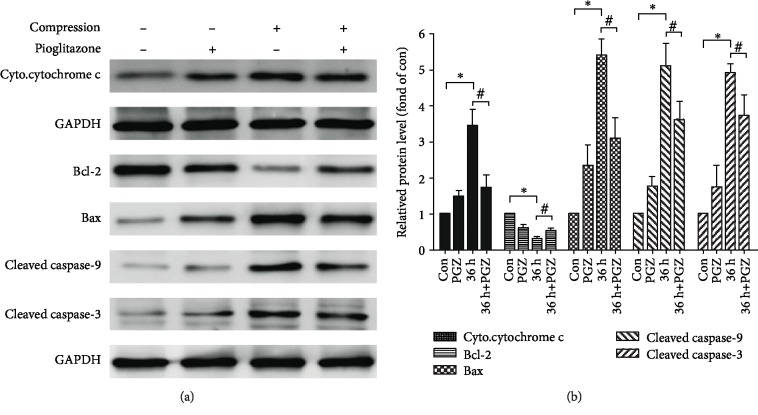
Effect of pioglitazone on mitochondrial apoptosis-related markers in NP-MSCs. (a) Representative western blotting results for cyto.cytochrome c, Bcl-2, Bax, cleaved caspase-9, and cleaved caspase-3 expression. (b) Quantitative analysis of Bax, cyto.cytochrome c, Bcl-2, cleaved caspase-9, and cleaved caspase-3 protein levels in NP-MSCs. The con group served as a control group. Data are presented as the mean ± SD of three independent experiments. ^∗^*P* < 0.05 versus the control group; ^#^*P* < 0.05 versus the compression group.

To investigate the effects of pioglitazone on compression-induced changes of the mitochondrial pathway apoptosis-related protein expression, we used western blotting to detect the expression of apoptosis-related proteins. The results revealed that the expression of the antiapoptotic protein Bcl-2 decreased, while that of the proapoptotic protein Bax increased in the compression group compared with the pioglitazone group. Also, we found that compression increased the release of cytochrome c into the cytosol. The expression levels of cleaved caspase-9 and cleaved caspase-3 of the compression group were higher than those of the control group. However, pioglitazone significantly reversed these effects (Figure 7(a)). Also, we have performed quantitative analysis of the protein levels (Figure 7(b)).

## 4. Discussion

In this research, we firstly investigated the effect that pioglitazone could protect NP-MSCs from compression-induced apoptosis and the possible molecular mechanisms. In the research, pioglitazone could reduce the effects of compression on NP-MSCs proliferation and cytotoxicity. Our results revealed that pioglitazone alleviated compression-induced NP-MSCs apoptosis via inhibition of oxidative stress and mitochondrion damage. Also, we found that pioglitazone alleviated the mitochondrial pathway apoptosis-related protein expression caused by compression.

Low back pain due to IVD degeneration increases the global economic burden and affects the quality of life, which has become a global health problem [1–3]. Many recent researches found that NP-MSCs existed naturally in the IVD and these NP-MSCs efficiently repaired NP tissue [6, 8, 32]. NP-MSCs play an important role in IVD endogenous repair, which differentiate into NP-like cells and stimulate disc cells maintaining IVD homeostasis [33]. Furthermore, NP-MSCs are better adapted to the harsh IVD microenvironment than exogenous MSCs such as tolerating hypoxic and hypertonic [7, 34]. In this research, we successfully isolated NP-MSCs from human degenerative IVD. Those cells, highly expressing surface markers CD73, CD90, and CD105 but less CD34 and HLA-DR, had the similar multilineage differentiation potential as BMSCs. Although endogenous NP-MSCs could be an attractive strategy for endogenous repair, it is hard to maintain the number of viable and functional NP-MSCs under an adverse IVD microenvironment.

Mechanical stress plays a critical role in the progress of IVD degeneration via IVD cell apoptosis [35]. We also reported that compression-induced NP-MSCs apoptosis contributed to IVD degeneration [12]. In this study, our results showed that compression had inhibitory effects on cell viability and proliferation. Also, it caused NP-MSCs apoptosis. Pioglitazone plays potential beneficial roles in inflammation, fat distribution, and lipid and protein metabolism [14–16]. A previous study reported that pioglitazone could attenuate fatty acid-induced oxidative stress and apoptosis in pancreatic cells [36]. Additionally, pioglitazone can also prevent H_2_O_2_-induced apoptosis of endothelial progenitor cells [37]. Zhang et al. also reported that pioglitazone significantly inhibited AGE-induced chondrocyte apoptosis and degeneration [20]. Interestingly, we found that pioglitazone alleviated the inhibitory effects of compression on cell viability. It also alleviated the inhibitory effects of compression on cell proliferation and cytotoxicity. Furthermore, pioglitazone rescued the breaking of skeleton structure and stress fibers induced by compression. The results of flow cytometry and TUNEL staining showed that pioglitazone had protective effects on compression-induced NP-MSCs apoptosis.

Oxidative stress, caused by high ROS, was widely considered a potent proapoptotic factor. Maintaining a balance between ROS generation and ROS scavenging is essential for the intracellular redox homeostasis. Mitochondria can produce ROS when they are damaged, which will further exacerbate the damage and cause the overproduction of ROS, then induce the occurrence of oxidative stress [38]. Recent studies have indicated that the establishment and progression of IVD degeneration are closely related to ROS products and oxidative stress. Oxidative stress not only accelerates extracellular matrix degradation of IVD but also causes cell apoptosis that leads to the decrease of the cells in the IVD microenvironment, all of the above contribute to IVD degeneration [39]. Ding et al. have reported that the mitochondrial apoptosis pathway participates in NP cell apoptosis induced by ROS overproduction [40]. Therefore, a therapeutic strategy targeting oxidative stress would provide a major method for treating IVD degeneration. Also, intracellular accumulated ROS resulted in releasing cytochrome c from mitochondria to the cytosol and eventually activating caspases, causing cell apoptosis [41–43]. In this study, we used DCFH-DA fluorescent probes to detect ROS production and found that pioglitazone can significantly reduce compression-induced ROS production. Also, the results of the MDA assay showed that pioglitazone alleviated compression-induced oxidative stress levels in NP-MSCs.

Mitochondrion dysfunction plays an important role in cell death [44]. Mitochondria, as a key regulator of cellular processes acting as cellular oxygen sensors [45], not only produce adenosine triphosphate (ATP) but also regulate cell proliferation and death [46]. Some evidence shows that cell apoptosis is closely connected with mitochondrial dysfunction, including MMP depolarization, enhanced mitochondrial permeability transition pore (mPTP) opening, and mitochondrial cristae disruption [47]. In recent years, Li et al. found that pioglitazone ameliorated palmitate-induced impairment of mitochondrial morphology and function in beta cells [48]. In addition, a literature from Shokrzadeh et al. reported that pioglitazone could reduce diabetic toxicity induced by streptozocin via improving mitochondrial function [49]. In this research, we found that compression induced the loss of MMP and damaged the number and structure of mitochondria. Pioglitazone significantly reversed these effects induced by compression. These results indicated that pioglitazone has protective roles in compression-induced mitochondrion damage.

The mitochondrial apoptosis pathway is caused by apoptotic signals, including mitochondrion dysfunction. Then, it leads to the loss of MMP and the release of cytochrome c into the cytosol. The release of cytochrome c is suppressed by the antiapoptotic protein Bcl-2 and stimulated by the proapoptotic member Bax, which activated the initiator of the intrinsic apoptotic pathway caspase-9. Then, it cleaved and activated the downstream factor, caspase-3, and triggered apoptosis [40, 50]. In this study, our results indicated that pioglitazone can significantly alleviate the compression-induced expression of cytochrome c, Bax, cleaved caspase-9, and caspase-3. Also, pioglitazone promoted the expression of antiapoptotic protein Bcl-2 compared with the compression group. Such results indicated that pioglitazone exerted a protective role in compression-induced cell apoptosis in NP-MSCs by inhibiting the mitochondrial apoptotic pathway.

Our results were highly reproducible. However, there were three limitations about this study. Firstly, we performed all experiments in vitro, and the conclusions may not be necessarily indicative of what occurs in vivo. Secondly, it is difficult to obtain healthy human NP tissue, so we used NP samples from patients of degenerative disc disease for this research. Thirdly, this study mainly focused on the protective effects of pioglitazone on compression-induced NP-MSCs apoptosis. We will further investigate the functional aspects of NP-MSCs from mesenchymal markers and multilineage differentiation, stem cell-related proteins, and genes in future studies. Also, we will use 3D-culture systems and a hypoxic condition to simulate IVD microenvironment in further studies.

## 5. Conclusions

In this research, the results indicated that pioglitazone alleviated compression-induced NP-MSC apoptosis by inhibiting oxidative stress and mitochondrial damage. The underlying molecular protective mechanism of pioglitazone on compression-induced apoptosis involves the mitochondrial pathway. Taken together, these findings may provide a valuable candidate for the treatment of IVD degeneration.

## Data Availability

The data used to support the findings of this study are available from the corresponding authors upon request.
